# Sacral Giant Cell Tumor Presenting as Low Back Pain in the Chiropractic Office: A Case Report

**DOI:** 10.7759/cureus.33262

**Published:** 2023-01-02

**Authors:** Eric C Chu, Robert J Trager, John S Shum, Kenny K Ng

**Affiliations:** 1 New York Chiropractic and Physiotherapy Centre, New York Medical Group, Kowloon, HKG; 2 Chiropractic, Connor Whole Health, University Hospitals Cleveland Medical Center, Cleveland, USA; 3 College of Chiropractic, Logan University, Chesterfield, USA; 4 Radiology, Hong Kong Advanced Imaging, Kowloon, HKG; 5 Medical Oncology, reVIVE Oncology and Cancer Centre, Mong Kok, HKG

**Keywords:** neoplasms, radiculopathy, sacrum, low back pain, giant cell tumors, chiropractic

## Abstract

Sacral giant cell tumors are a rare cause of low back pain and may be challenging to identify via routine clinical examination and radiography. A 47-year-old woman presented to a chiropractor with a one-month history of worsening low back pain with radiation to the posterior thighs, worsened with ambulation, and used a cane to walk. She previously saw an orthopedic surgeon and was diagnosed with lumbar spondylosis, having tried anti-inflammatory medications, exercises, and acupuncture without success. The chiropractor ordered lumbar magnetic resonance imaging which revealed an aggressive sacral lesion and referred the patient to an oncologist. The oncologist performed positron emission tomography/computed tomography and biopsy, confirming a sacral giant cell tumor. A surgical team recommended tumor resection, lumbosacral fusion, radiotherapy, and zoledronic acid infusion. Sacral giant cell tumors are rare and may be challenging to identify via routine radiography. These tumors are an important differential to consider for patients with unexplained lumbosacral symptoms unresponsive to care.

## Introduction

Giant cell tumors (GCTs) of bone are locally aggressive primary tumors that rarely undergo malignant transformation [1,2]. GCTs most often occur in the knee and distal radius, with the sacrum being the third most common site [1]. Involvement of the sacrum may cause low back pain and sciatica, and mimic more common musculoskeletal sources of pain.

Most patients with GCT are diagnosed between the ages of 20 and 40 [1], and there is a slight predilection to affect females [1,3]. GCT is more common in China compared to other countries, with an annual incidence of up to 2.6 cases per million persons [4]. GCT of the sacrum typically presents with low back or sacral pain and may include hip, buttocks, or lower extremity pain [1,5]. Patients may also present with neurological symptoms due to sacral nerve involvement, such as lower extremity weakness, or cauda equina-related symptoms such as bowel or bladder dysfunction [1,6-8].

Sacral GCTs are challenging to diagnose as they are often clinically silent early on and may not be identified until they reach a large size [9]. GCTs may be missed by plain radiographs, in part due to their location being obscured by superimposed bowel gas [1]. Further, when symptoms are present, they are often ascribed to the more common radicular sciatica rather than a neoplastic etiology [1].

As chiropractors often treat low back pain, it is possible that patients with undiagnosed sacral GCT could present to these providers. However, a search of PubMed, Google Scholar, and the Index to Chiropractic Literature on December 16, 2022, using the search terms “chiropractor” and “giant cell tumor,” and hand-searching of a review paper [10] revealed only one similar case in which a patient with sacral GCT being treated in an orthopedic oncology department reported previously seeing a chiropractor [5]. One other case described a patient with a cervical spine GCT who had presented to a chiropractor [11]. To our knowledge, identification of a sacral GCT in the chiropractic office has not been previously reported.

Considering that sacral GCTs may cause low back pain and are difficult to diagnose, we present the case of a woman with sacral GCT which was initially missed via radiography and then identified after presenting to a chiropractor who ordered magnetic resonance imaging (MRI).

## Case presentation

A 47-year-old woman presented to a chiropractor with an exacerbation of frequent pain at the lumbosacral and coccygeal regions, with radiation to the proximal posterior thighs bilaterally, rated 8/10 on the numeric pain rating scale. The patient reported a two-year history of mild, localized low back pain, which had gradually worsened over the past four weeks without a specific injury. She noted being unable to sit, stand, or walk for over 15 minutes, and recently began using a cane to help with ambulation. She noted the pain could be aggravated by coughing or sneezing and lying supine, and her sleep quality was accordingly poor. She endorsed fatigue but denied having any bowel or bladder disturbances, nausea, vomiting, or recent change in weight. She was a non-smoker and did not drink alcohol.

**Figure 1:**
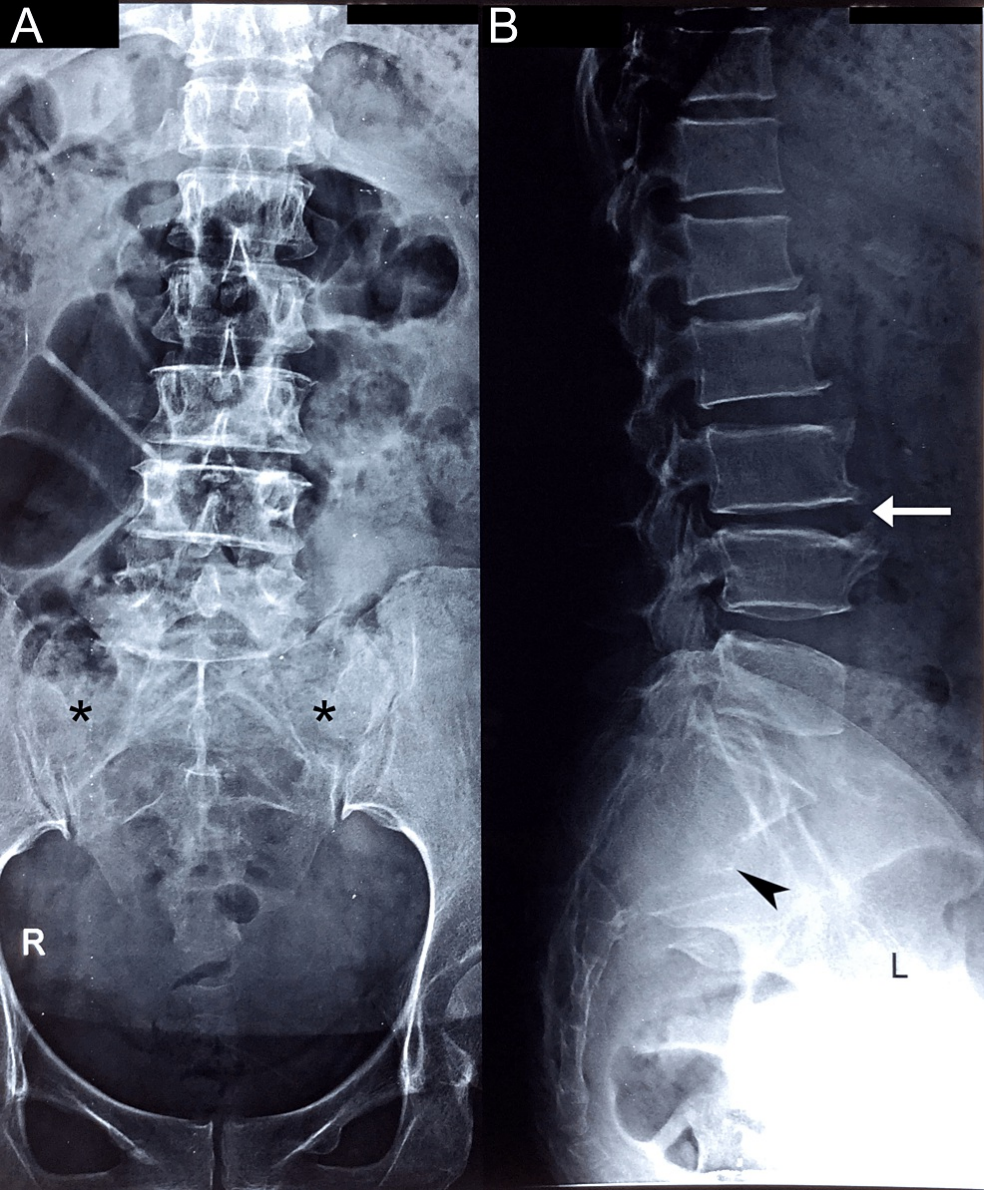
Lumbar radiographs, anteroposterior view (A) and lateral view (B). These radiographs were originally interpreted as only showing marginal osteophytes and disc space narrowing at L3-L4, which is best seen on the lateral view (arrow). However, after advanced imaging, it was apparent that there were subtle signs of abnormality that were not identified earlier, including a slight lucency of the sacral alae (*) and disruption of the anterior cortex of the sacrum (arrowhead). These findings are partially obscured by bowel gas in image A and overlapping anatomy of the pelvis in image B.

She initially tried over-the-counter nonsteroidal anti-inflammatory drugs (NSAIDs) which did not relieve her symptoms. She then consulted with an orthopedic surgeon who ordered lumbar radiography. The radiography report mentioned degenerative changes but did not mention any suspicious findings in the sacral region (Figure 1). The orthopedic surgeon diagnosed the patient with lumbar spondylosis and prescribed NSAIDs (etoricoxib and celecoxib), prescribed home exercises, and referred her for acupuncture. None of these therapies provided the patient with relief.

On physical examination by the chiropractor, the patient reported tenderness along the lumbar paraspinal musculature. Palpation revealed motion restriction and tenderness at the T7-T8 and T12-L1 levels, as well as with pressure over the upper sacrum. The examination also revealed hypertonicity at the iliopsoas, rectus femoris, and erector spinae bilaterally. The patient’s lumbar spine range of motion was restricted with an increase in pain at 15° extension (normal 20°-35°). Straight leg raising did not reproduce her symptoms. Her neurological evaluation revealed 4/5 strength (Medical Research Council Scale) of hip abduction bilaterally and her Achilles muscle stretch reflexes were 1+ bilaterally. Sensation to light touch was intact.

The chiropractor’s initial differential diagnosis was (1) lumbosacral radiculopathy based on the lumbosacral pain with radiation and weakness, followed by (2) coccydynia, based on positional tenderness at the sacral region. However, considering the patient’s worsening clinical picture and lack of relief with previous treatments, the chiropractor ordered a lumbar MRI on the day of the first consultation, which was performed two days later. Initially, prior to receiving the MRI results, the chiropractor performed a trial of manual lumbar spinal manipulation, avoiding pressure over the sacrum given the tenderness in this region. This treatment was well-tolerated and provided the patient with transient relief.

**Figure 2:**
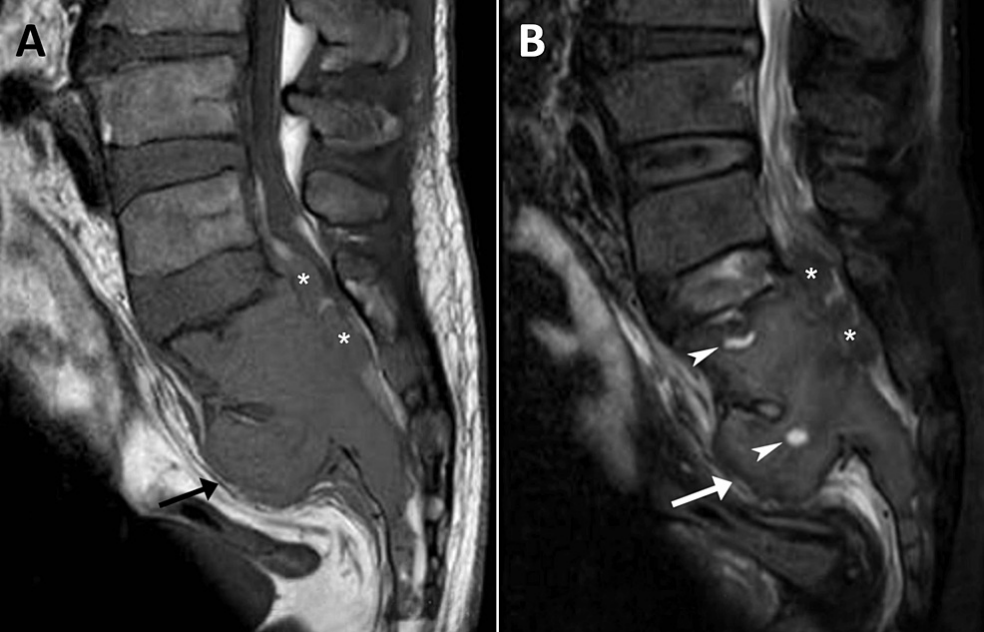
Lumbar spine magnetic resonance imaging, T1-weighted (A) and T2-weighted (B) sagittal views. There is a large, expansile bone lesion in the sacral ala and bodies S1-S3, which is mildly hypointense on T1 and intermediate signal intensity on T2, with small areas of hyperintensity on the T2-weighted view (B, arrowheads), suggestive of cystic spaces. There is anterior cortical destruction at S2 with soft-tissue penetration into the pre-sacral space (arrow). Also noted is cortical destruction and extension into the sacral spinal canal at the S1 and S2 levels (*).

The patient’s lumbar MRI revealed an irregular bone lesion infiltrating the upper sacrum from S1-S3, measuring 6.4 × 4.7 cm on the sagittal view, in addition to evidence of previously identified degenerative changes (Figure 2). Accordingly, the working diagnosis was now revised to a primary sacral tumor, such as a sacral GCT, chordoma, or sarcoma, while myeloma was also possible. As MRI findings were concerning for neoplasm, the chiropractor referred the patient to an oncologist for further evaluation, whom she saw the following day.

**Figure 3:**
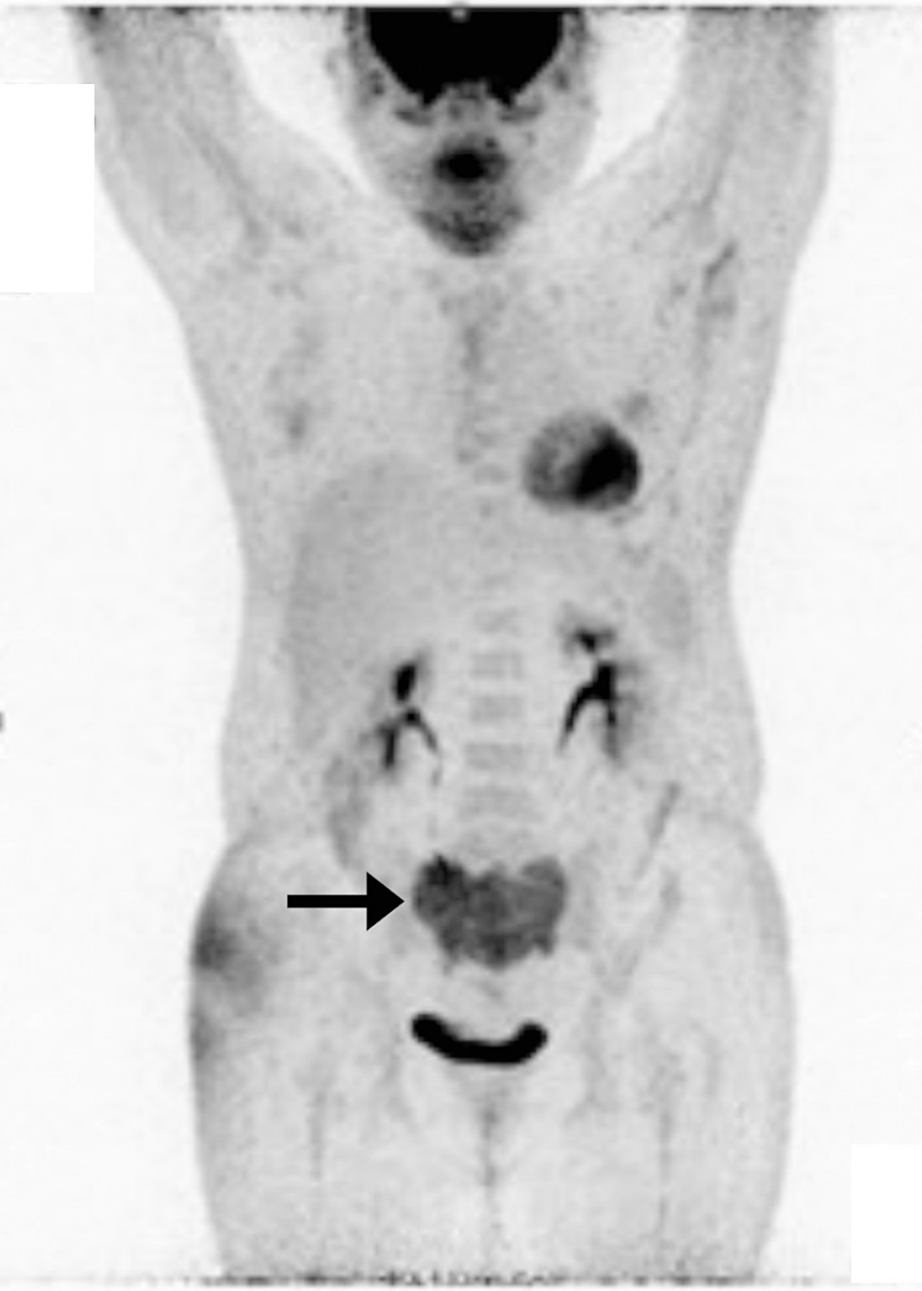
Fluorodeoxyglucose whole-body positron emission tomography/computed tomography. Increased fluorodeoxyglucose uptake is evident within the sacrum (arrow), with a standardized uptake value of 9.82.

The oncologist ordered positron emission tomography/computed tomography (PET/CT) as a screening test for possible metastasis and further characterize the sacral lesion, as well as CT-guided biopsy. Fluorodeoxyglucose whole-body PET/CT revealed a destructive, hypermetabolic sacral bone lesion with encasement of the sacral canal at the S1-S3 levels, and involvement of the presacral soft tissue (Figure 3). There was no evidence of hypermetabolic skeletal, nodal, or distant metastases. Considering the PET/CT showed no signs of metastasis, the sacral lesion was deemed to represent a primary benign sacral bone tumor.

**Figure 4:**
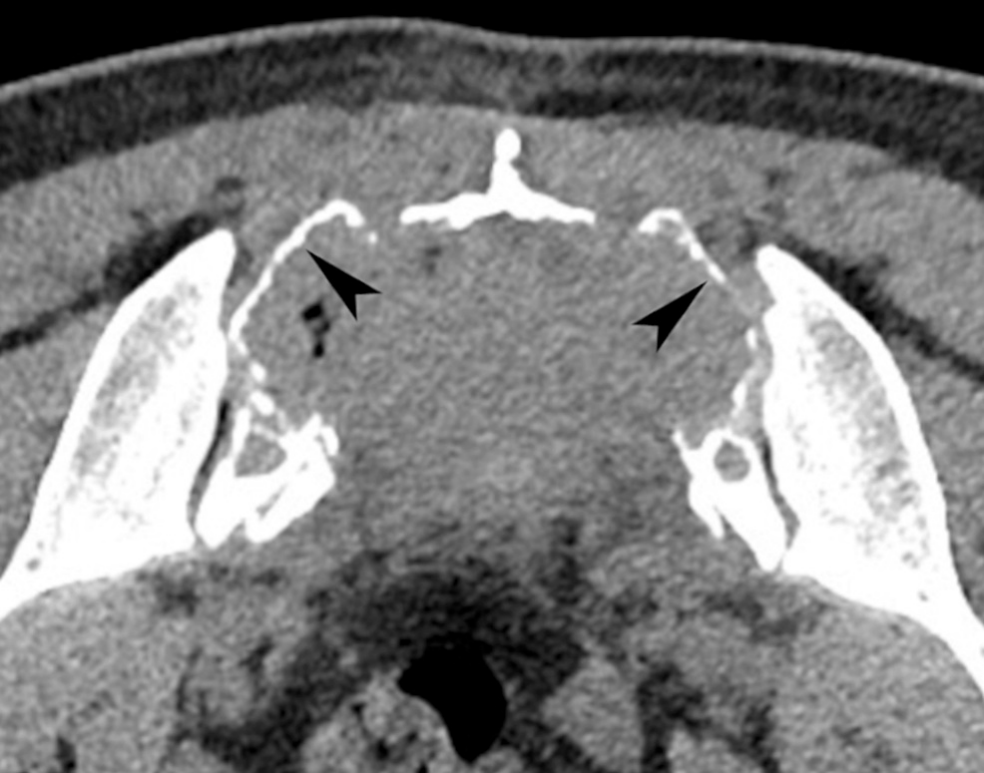
Axial computed tomography section through the first sacral segment. This reveals a lytic, expansile mass with soft-tissue attenuation, spanning the sacral body and alae bilaterally, with thinning but no sclerosis of the cortical bone (arrowheads).

CT-guided biopsy was performed using a left posterior approach, targeting the S1-S3 levels. The CT scan revealed a large lytic sacral mass with cortical thinning, a non-sclerotic border, and no signs of pathologic fracture (Figure 4). Macroscopically, the biopsy yielded seven cores of soft tan-colored to brownish and focally congested tissue.

**Figure 5:**
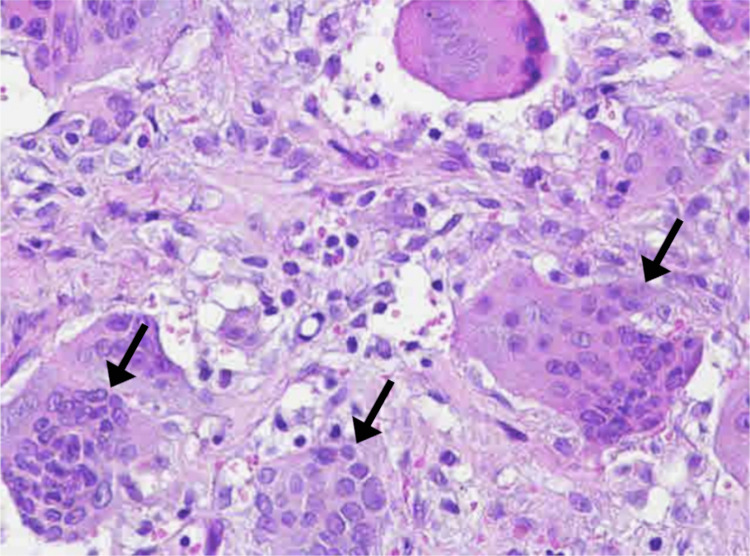
Hematoxylin and eosin section showing multinucleated giant cells (arrows) with interposed mononuclear cells. 400× magnification.

Histopathology of the mass revealed an indolent giant cell-rich bone lesion, consisting of abundant osteoclast-like multinucleated giant cells associated with bland-looking spindly fibroblast-like mononuclear cells, young capillaries, and focal stromal hemorrhage, findings typical for GCT (Figure 5). There was no evidence of suppuration, granulomatous inflammation, chondroid/epithelial elements, coagulative necrosis, high mitotic activity, significant cytological atypia, dysplasia, or overt malignancy. Following the CT-guided biopsy and histopathological analysis, the diagnosis was almost certain as a sacral GCT. The oncologist also ordered serum calcium (2.50 mmol/L) and parathyroid hormone (24 pg/mL) which were within normal limits. While brown tumors of hyperparathyroidism also contain giant cells, a brown tumor was excluded after testing of serum calcium and parathyroid hormone were normal, making GCT the confirmed diagnosis.

Based on the conclusive findings of the imaging, biopsy, and laboratory testing, the oncologist referred the patient to a spine surgery team. After discussion with the surgical team, the patient consented to an en-bloc resection of the sacral tumor with a lumbosacral fusion with instrumentation, followed by radiotherapy and zoledronic acid infusion. Details regarding the patient’s surgery and postoperative course were unavailable from the patient and treating spine surgery department upon request. Written informed consent was obtained from the patient to publish her case and any accompanying images.

## Discussion

This case illustrates, to our knowledge, the first patient with sacral GCT identified by a chiropractor. This case reinforces that sacral GCTs are challenging to identify, and because of this, such patients may seek a chiropractic provider due to having symptoms of low back pain that appear musculoskeletal in origin.

Providers should be aware of the optimal diagnostic algorithm for sacral GCT given the pitfalls in their diagnosis. For example, symptoms mimic more common forms of low back pain, there may be no overt radiographic features [1], and neurologic deficits only may arise late in tumor growth [9]. Therefore, providers should conduct a thorough history and examination of patients [1,2], as well as recognize when patients are worsening despite conservative care. Further, previous radiographs should be scrutinized, when available, including areas of overlapping anatomy and bowel gas around the sacrum.

When clinical features are suggestive of a primary sacral tumor such as sacral GCT, CT and MRI may be helpful to assess the extent of the tumor [1,2]. Scintigraphy or PET/CT may be used to evaluate for metastasis [1]. Ultimately, a biopsy is needed to distinguish sacral GCT from other sacral tumors and confirm the diagnosis [1,2]. In the current case, additional laboratory testing of serum calcium and parathyroid hormone helped further solidify the diagnosis.

There is currently no consensus on the treatment of sacral GCTs [6]. Non-surgical treatments such as denosumab, angioembolization, and radiotherapy have shown some promise [8], but they also come with risks and uncertainty due to limited research [3]. Radiotherapy has been reported to cause sarcomatous transformation of GCT, and, as such, is usually avoided in isolation [3].

Surgical options likewise carry significant risks due to the location and potential large size of sacral GCTs. In particular, spinal/pelvic instability, nerve root injury, and risk of infection are potential drawbacks [1,6]. However, en-bloc resection is recommended whenever possible as the treatment of choice for spinal GCT [7]. Given the limited available treatments and potential drawbacks to surgical procedures for sacral GCT, patients may ultimately reach a decision after discussing options with their surgical team.

## Conclusions

This case highlights a woman with low back pain caused by sacral GCT which was initially missed by radiography and then identified by a chiropractor who ordered MRI. As GCTs of the sacrum may cause chronic low back pain and can be challenging to visualize with plain radiography, patients could present to chiropractors unaware that they have a sacral tumor. Sacral GCTs should be diagnosed and characterized with advanced imaging and biopsy to be differentiated from other primary sacral tumors. As there is no consensus strategy on the treatment of sacral GCTs, patients may make an informed decision regarding treatment in conjunction with their surgical and oncologic providers’ recommendations.
